# Early-life exposure to antibiotics increases the risk of myopia: A retrospective cohort study

**DOI:** 10.37796/2211-8039.1666

**Published:** 2026-06-01

**Authors:** Peng-Tai Tien, Chang-Ching Wei, Sheng-Chun Lin, Ping Ping Meng, Yu-Te Huang, Jamie Jiin-Yi Chen, Ming-Yen Wu, Ying-Hsiu Shih, Tzu-Ju Hsu, Fuu-Jen Tsai, Yao-Chien Wang, Hui-Ju Lin, Lei Wan

**Affiliations:** a Eye Center, China Medical University Hospital, China Medical University, Taichung 40447, Taiwan; b School of Medicine, China Medical University, Taichung 40447, Taiwan; c Division of Allergy, Immunology and Rheumatology, Department of Pediatrics, Children’s Hospital, China Medical University Hospital, Taichung 40447, Taiwan; d College of Medicine, China Medical University, Taichung 40447, Taiwan; e Graduate Institute of Biomedical Sciences, China Medical University, Taichung 406040, Taiwan; f Management Office for Health Data, China Medical University Hospital, Taichung 40447, Taiwan; g Department of Emergency Medicine, Taichung Tzu Chi Hospital, Taichung 42743, Taiwan; h Department of Medical Research, China Medical University Hospital, China Medical University, Taichung, Taiwan; i School of Chinese Medicine, College of Chinese Medicine, China Medical University, Taichung, Taiwan; j China Medical University Children’s Hospital, Taichung, Taiwan; k Department of Medical Genetics, China Medical University Hospital, Taichung, Taiwan; l Department of Medical Laboratory Science and Biotechnology, Asia University, Taichung 40402, Taiwan; m Department of Obstetrics and Gynecology, China Medical University Hospital, Taichung 40447, Taiwan

**Keywords:** Myopia, Antibiotic exposure, Gut microbiome, Dysbiosis, Early-life exposure, Pediatric ophthalmology

## Abstract

**Background:**

The prevalence of myopia is increasing worldwide and is projected to affect nearly half of the global population by 2050. Although genetic susceptibility plays an important role, early-life environmental exposures are increasingly recognized as modifiable contributors to myopia development. Antibiotics are frequently prescribed during infancy and early childhood and can alter gut microbiota composition, which has been implicated in ocular disorders.

**Methods:**

We conducted a retrospective cohort study using data from a national health insurance database. Children aged three years or younger who received systemic antibiotics were included, while those with pre-existing myopia or retinopathy were excluded. Antibiotic-exposed children were matched with non-exposed controls by age, sex, and relevant comorbidities. Cox proportional hazards regression models were used to estimate adjusted hazard ratios (aHRs) and 95% confidence intervals (CIs) for incident myopia.

**Results:**

Early-life antibiotic exposure was associated with a significantly increased risk of myopia compared with non-exposed controls (aHR = 1.03; 95% CI: 1.02–1.04), accompanied by a higher cumulative incidence of myopia (log-rank test, p < 0.001). The elevated risk was observed in both females (incidence rate [IR] = 66.98 vs. 64.10; aHR = 1.05; 95% CI: 1.04–1.07) and males (IR = 61.53 vs. 59.66; aHR = 1.04; 95% CI: 1.02–1.05). Urbanization level, parental monthly income, and comorbidities were independently associated with antibiotic exposure. The increased risk of myopia persisted irrespective of antibiotic treatment duration (<11 days; aHR = 1.19; 95% CI: 1.15–1.23) or cumulative dose (defined daily dose <2833; aHR = 1.31; 95% CI: 1.28–1.33).

**Conclusions:**

Early-life exposure to antibiotics is associated with a higher incidence of myopia in children. These findings underscore the importance of cautious and judicious antibiotics prescribing during early childhood to mitigate potential long-term ocular health risks.

## 1. Introduction

Myopia is the most frequent cause of correctable visual impairment worldwide and its prevalence is alarmingly increasing over the years as a global public health burden [1,2]. Myopia is expected to affect nearly 50% of the global population by 2050, and approximately 20% of the myopic population will develop high myopia [3]. In addition to the inconvenience of wearing eyeglasses or contact lenses to achieve optimal visual acuity and the associated lifelong economic burden, high myopia is associated with serious pathological complications, including retinal detachment, myopic macular degeneration, early-onset cataracts, and glaucoma [1,4]. Recent research indicates that an earlier age of myopia onset is a significant risk factor for the development of high myopia later in life [2–4].

Myopia is epidemic in East Asia, reaching 80% by 18 years of age. Taiwanese schoolchildren have the highest prevalence of myopia among all schoolchildren in the world [5–7]. According to nationwide surveys conducted among Taiwanese schoolchildren between 1983 and 2017, the prevalence of myopia increased from 5.4% to 25.4% for 7-year-olds and from 30.7% to 76.7% for 12-year-olds [5]. Moreover, the prevalence of myopia has reached a plateau of 92.9% and 90.3% among junior and senior high school students, respectively. The data demonstrate a clear trend of an increasing rate of high myopia associated with earlier onset of myopia [5]. Identifying modifiable risk factors for myopia is particularly important and urgent in Taiwan.

Despite the widespread occurrence, the exact cause of myopia remains unclear. Current evidence suggests that myopia is a complex trait influenced by a combination of environmental exposures, lifestyle factors, and genetic variation [8]. Reducing the burden of myopia in the population requires identification of modifiable risk factors, such as environmental factors, and assessment of the effectiveness of risk-reduction measures. In addition, understanding environmental risk factors that are amenable to policy changes is essential for the effective prevention of myopia onset and progression.

Antibiotics are commonly prescribed to infants and toddlers because they are susceptible to infections [9]. Antibiotic-induced changes in microbial diversity and the dysbiosis of the gut microbiome due to exposure to antibiotics in early life have been associated with various conditions, including inflammatory bowel disease, type 2 diabetes, allergic disorders, and ocular diseases [10]. Research has shown that different types and durations of antibiotics may have variable influences on microbial composition and even with short-term antibiotic use [11,12]. Therefore, there is growing interest in the potential causal relationship between antibiotic-related dysbiosis in early life and the subsequent risk of myopia. We conducted a population-based study in Taiwan, a region with the highest prevalence of myopia among schoolchildren worldwide, to investigate the association between antibiotic use in children aged three years or younger and their subsequent risk of myopia.

## 2. Materials and methods

### 2.1. Data source

The National Health Insurance Research Database (NHIRD) provides comprehensive medical information for nearly the entire population of Taiwan. The database provides longitudinal healthcare data suitable for population-based epidemiological research. In this study, NHIRD population data were utilized, and disease codes were classified based on the International Classification of Diseases, 9th and 10th editions, clinically modified (ICD-9-CM and ICD-10-CM). This work was approved by the Institutional Review Board of the Research Ethics Committee of the Affiliated Hospital of China Medical University, Taiwan (CMUH109-REC2-031(CR-2)).

### 2.2. Study population and outcome

The selection of study subjects from a random sample of 29,051,112 individuals diagnosed with myopia between 2000 and 2018 is presented in Supplementary Figure 1 (https://www.biomedicinej.com/cgi/editor.cgi?article=1666&window=additional_files&context=biomedicine). To ensure the accuracy of the myopia diagnosis, myopia was defined as having at least two outpatient visits or one admission record with corresponding disease codes (ICD-9-CM:367.1; ICD-10-CM: H52.1). Patients older than 3 years at first antibiotic use (n = 28,203,130), those with prior neonatal conditions, cataracts, or other retinopathies before the index date (n = 172,525), and those with a prior diagnosis of the outcome (n = 804) were excluded from the study. The case and control groups were matched in a 1:1 ratio using propensity score matching, based on factors such as sex, age, urbanization, monthly income, and comorbidities. In total, 317,164 patients with myopia were included in the analysis. Patients with myopia were then divided into two groups: those who did not use antibiotics (n = 158,582) and those who used antibiotics (n = 158,582).

### 2.3. Comorbidities

These comorbidities included allergic rhinitis (ICD-9-CM: 477; ICD-10-CM: J30), pneumonia (ICD-9-CM: 481, 482.0–482.4, 482.81–482.83, 483.89, 482.9, 483.0; ICD-10-CM: J13–J15), bronchitis (ICD-9-CM: 464.0, 464.1, 464.20, 466; ICD-10-CM: J04, J20, J21), sinusitis (ICD-9-CM: 461; ICD-10-CM: J01), acute otitis media (ICD-9-CM: 381.0–381.4; ICD-10-CM: H65), ear cellulitis (ICD-9-CM: 380.1, 380.2; ICD-10-CM: H60), pharyngitis (ICD-9-CM: 034.0, 462; ICD-10-CM: J02), tonsillitis (ICD-9-CM: 034.0, 463, 474; ICD-10-CM: J03, J35), laryngitis (ICD-9-CM: 464.0, 464.21, 464.3, 464.4, 465; ICD-10-CM: J05, J06), orbital cellulitis (ICD-9-CM: 376; ICD-10-CM: H05), hordeolum (ICD-9-CM: 373.1, 373.2; ICD-10-CM: H00), gastroenteritis (ICD-9-CM: 009; ICD-10-CM: A09), cellulitis (ICD-9-CM: 528.0, 528.2, 528.3, 528.9, 681, 682; ICD-10-CM: K12, L03), urinary tract infection (ICD-9-CM: 595, 599.0; ICD-10-CM: N30, N39.0), sepsis (ICD-9-CM: 038; ICD-10-CM: A40, A41), food allergy (ICD-9-CM: 693.1, 995.6, V15.01-V15.05; ICD-10-CM: L27.2, T78.01XA-T78.09XA, Z91.01), allergic conjunctivitis (ICD-9-CM: 372.05, 372.10, 372.14; ICD-10-CM: H10.1, H10.40, H10.41, H10.45), asthma (ICD-9-CM: 493; ICD-10-CM: J44, J45.2-J45.5, J45.90, J45.991, J45.998) and atopic dermatitis (ICD-9-CM: 691; ICD-10-CM: L20, L22). Comorbidities were defined as at least two outpatient visits or one admission record. However, cases with prematurity (ICD-9-CM: 765; ICD-10-CM: P07), neonatal problems (ICD-9-CM: 760–779; ICD-10-CM: A33, A48.51, J84.83, J84.84, P00–P08, P10–P28, P29.0, P29.1, P29.2, P29.4, P29.8, P29.9, P35–P36, Q86, R78.81), cataract (ICD-9-CM: 366; ICD-10-CM: E08.36, E09.36, E10.36, E11.36, E13.36, H25, H26, H28), or other retinopathies (ICD-9-CM: 362; ICD-10-CM: E11.31–E11.35, G45.3, H31.1, H34–H36) prior to the index date were excluded from the study (Supplementary Figure 1 (https://www.biomedicinej.com/cgi/editor.cgi?article=1666&window=additional_files&context=biomedicine)).

### 2.4. Statistical analysis

In the analysis, categorical variables such as sex, urbanization, monthly income, and comorbidities were presented as events and percentages. Chi-square tests were used to assess the differences between categorical variables of the case and control groups. Age and follow-up time were presented as mean ± standard deviation, and independent t-tests were used to examine the differences between continuous variables between groups. The Cox regression model was utilized to calculate the incidence rate and hazard ratio of myopia. Additionally, the incidence rate and hazard ratio of myopia in the case group compared to the control group were determined. The hazard ratio was adjusted for sex, age, urbanization, monthly income, and comorbidities. The cumulative incidence rate curve of myopia was calculated using the Kaplan-Meier method, and the p-value was estimated using the log-rank test. A two-sided p-value less than 0.05 was considered statistically significant. The analyses were performed using SAS software, version 9.4 (SAS Institute Inc., Cary, NC), and the graph was generated using RStudio.

## 3. Results

The baseline characteristics of patients who used antibiotics compared to those who did not are presented in Table 1. After 1:1 propensity score matching, the number of patients in the antibiotics and non-antibiotics groups was 158,582 each. Both the case and the control groups had more female participants, accounting for 52.12% and 51.26%, respectively. The average patient age in both groups was 1.54 (±0.73) and 1.57 (±0.75) years, respectively. The most prevalent comorbidity among the participants was laryngitis, followed by bronchitis.

**Fig. 1:**
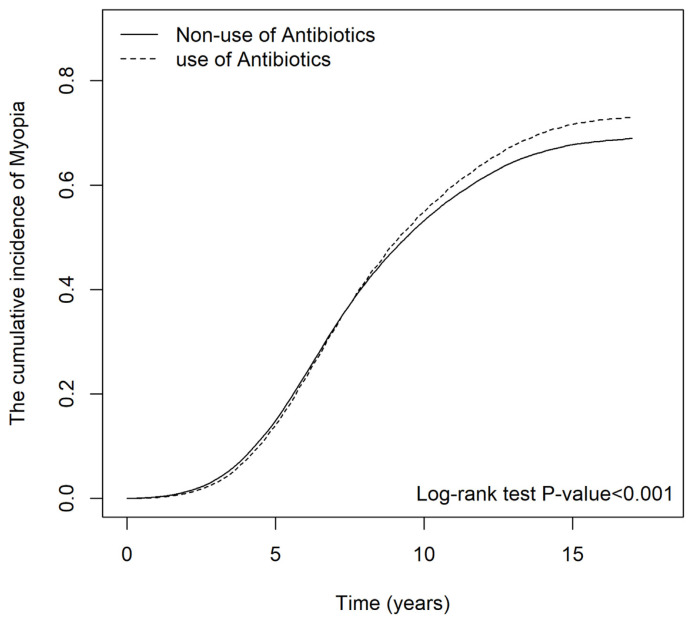
The Kaplan-Meier curves depict the cumulative incidence rate of myopia during the follow-up period for the groups that used and did not use antibiotics.

Individuals with early-life antibiotic exposure had a significantly higher risk of myopia compared to the control group (Table 2; aHR = 1.03, 95% CI = 1.02–1.04). This is supported by Fig. 1, which shows a higher cumulative incidence rate of myopia in the case group than in the control group (p < 0.001; log-rank test). Myopia risk was significantly lower in males (Table 2; aHR = 0.93, 95% CI = 0.92–0.94) than in females. Moreover, comorbidities were associated with a significantly higher risk of myopia. These comorbidities included allergic rhinitis (aHR = 1.04, 95% CI = 1.02–1.06), gastroenteritis (aHR = 1.03, 95% CI = 1.02–1.05), cellulitis (aHR = 1.07, 95% CI = 1.03–1.12), urinary tract infection (aHR = 1.12, 95% CI = 1.08–1.16), allergic conjunctivitis (aHR = 1.09, 95% CI = 1.07–1.12), and asthma (aHR = 1.08, 95% CI = 1.05–1.11) (Table 2). To further strengthen the effect of antibiotic exposure on the risk of myopia, we analyzed the myopia risk without propensity score matching. The results are shown in Supplementary Tables 1 and 2 (https://www.biomedicinej.com/cgi/editor.cgi?article=1666&window=additional_files&context=biomedicine). Individuals who had early-life antibiotic exposure had a significantly higher risk of myopia compared to the control group (Supplementary Table 2 (https://www.biomedicinej.com/cgi/editor.cgi?article=1666&window=additional_files&context=biomedicine); aHR = 1.14, 95% CI = 1.13–1.15).

In both females (IR = 66.98 vs. 64.10; aHR = 1.05; 95% CI = 1.04–1.07) and males (IR = 61.53 vs. 59.66; aHR = 1.04; 95% CI = 1.02–1.05), the risk for myopia was significantly higher in the case group than in the control group (Table 3). Furthermore, considering other factors, such as urbanization and monthly income, the risk of myopia in the case group remained significantly higher than that in the control group. Among the patients without comorbidities, the risk of myopia was significantly higher in the case group than in the control group (Table 3). Comorbidities including allergic rhinitis (IR = 64.14 vs. 61.17; aHR = 1.05; 95% CI = 1.04–1.06), pneumonia (IR = 64.23 vs. 61.80; aHR = 1.04; 95% CI = 1.03–1.05), bronchitis (IR = 67.09 vs. 58.57; aHR = 1.15; 95% CI = 1.13–1.17), sinusitis (IR = 67.11 vs. 62.46; aHR = 1.08; 95% CI = 1.07–1.09), acute otitis media (IR = 64.17 vs. 61.74; aHR = 1.04; 95% CI = 1.03–1.05), ear cellulitis (IR = 64.27 vs. 61.72; aHR = 1.05; 95% CI = 1.03–1.06), pharyngitis (IR = 65.96 vs. 61.28; aHR = 1.08; 95% CI = 1.07–1.09), tonsillitis (IR = 64.69 vs. 60.12; aHR = 1.08; 95% CI = 1.07–1.09), laryngitis (IR = 73.19 vs. 54.59; aHR = 1.35; 95% CI = 1.32–1.39), hordeolum (IR = 64.21 vs. 61.63; aHR = 1.05; 95% CI = 1.04–1.06), gastroenteritis (IR = 64.22 vs. 60.71; aHR = 1.06; 95% CI = 1.05–1.07), cellulitis (IR = 64.13 vs. 61.71; aHR = 1.04; 95% CI = 1.03–1.05), urinary tract infection (IR = 63.99 vs. 61.69; aHR = 1.04; 95% CI = 1.03–1.05), allergic conjunctivitis (IR = 64.09 vs. 61.21; aHR = 1.05; 95% CI = 1.04–1.06), asthma (IR = 63.76 vs. 61.35; aHR = 1.04; 95% CI = 1.03–1.05), and atopic dermatitis (IR = 66.49 vs. 62.38; aHR = 1.07; 95% CI = 1.06–1.08), all showed significantly higher risk of myopia in the case group than in the control group (Table 3). Among patients with urinary tract infection, the risk of myopia was significantly higher in the case group than in the control group (IR = 73.18 vs. 66.56; aHR = 1.11; 95% CI = 1.04–1.19) (Table 3).

Table 4 presents the risk of myopia among patients based on the duration of antibiotics use, categorized as < 11 days, 11–80 days, and ≥80 days. The results show that the risk of myopia was significantly higher in patients using antibiotics for <11 days (aHR = 1.19, 95% CI = 1.15–1.23), 11–80 days (aHR = 1.02, 95% CI = 1.01–1.04), and ≥80 days (aHR = 1.07, 95% CI = 1.05–1.08) compared to those who did not use antibiotics. We also analyzed the frequency of antibiotics use (Supplementary Table 3 (https://www.biomedicinej.com/cgi/editor.cgi?article=1666&window=additional_files&context=biomedicine)). We found that penicillins with extended spectrum (32.5%) and first-generation cephalosporins (26.7%) were most frequently used. We found that use of extended-spectrum penicillins for <9 days (aHR = 1.15, 95% CI = 1.11–1.20) and 9–43 days (aHR = 1.01, 95% CI = 1.00–1.03) was associated with a significantly higher risk of myopia. Patients who used first-generation cephalosporins for ≥32 days (aHR = 1.12, 95% CI = 1.10–1.14) had a significantly higher risk of myopia. All other antibiotics showed the same trend of increasing risk of myopia when administered for <8 days (aHR = 1.19, 95% CI = 1.07–1.33), 8–23 days (aHR = 1.19, 95% CI = 1.15–1.22), or ≥23 days (aHR = 1.35, 95% CI = 1.30–1.41).

The effect of the defined daily dose (DDD) of antibiotics on the risk of myopia was also determined. As presented in Table 5, the DDD for any antibiotics was categorized into <2833 (aHR = 1.31, 95% CI = 1.28–1.33) and 2833–6083 (aHR = 1.11, 95% CI = 1.09–1.13), both of which increased the risk of myopia. The DDD of penicillins with extended spectrum categorized into <4042 (aHR = 1.22, 95% CI = 1.19–1.24) and 4042–7833 (aHR = 1.06, 95% CI = 1.04–1.08) increased the risk of myopia. For first-generation cephalosporins, <5625 (aHR = 1.26, 95% CI = 1.24–1.29) and 5625–10134 (aHR = 1.07, 95% CI = 1.05–1.09) increased the risk of myopia. All other antibiotics were also associated with a higher risk of myopia.

## 4. Discussion

The underlying cause of myopia is unknown. Genetic and environmental factors play significant roles in myopia incidence. Moreover, the gut-retina axis has recently emerged as a potential biological factor contributing to several ocular diseases. We found that exposure to antibiotics before the age of 3 years increased the risk of myopia in children compared with those not exposed. These results suggest that dysbiosis due to antibiotic exposure may promote the development of myopia.

There are numerous indications of a potential link between the gut microbiota and the development of myopia. (1) Type 1 diabetes, uveitis, and systemic lupus erythematosus (SLE) are associated with an increased incidence of myopia [13]. Moreover, gut microbiota dysbiosis is associated with autoimmune diseases, including type 1 diabetes [14], uveitis [15], and SLE [16]. (2) Gut dysbiosis is linked to numerous allergic diseases, including asthma [17], atopic dermatitis [18], and allergic rhinitis [19]. Additionally, conjunctival dysbiosis is associated with allergic conjunctivitis [20] which can be managed by administering probiotics [21]. Risk factors for myopia include asthma, atopic dermatitis, allergic rhinitis, and allergic conjunctivitis [22]. (3) Air pollution also causes dysbiosis in the gut microbiome [23]. Greater exposure to particulate matter with a diameter less than 2.5 μm (PM_2.5_) increases myopia prevalence [24]. (4) Parental myopia is another significant risk factor and predictor of myopia in children. However, only a small proportion of myopic cases can be directly linked to specific genetic factors. It has been hypothesized that the interaction between genes and environment plays a significant role in the pathogenesis of myopia [25]. Parental dietary habits are known to influence the gut microbiota of the offspring [26], which may increase the risk of myopia. (5) Lactoferrin from bovine milk retards the progression of myopia in mice by reducing eye inflammation [27] and its oral administration modifies the gut microbiota and inflammatory responses [28,29]. Xiao et al. recently discovered that *Proteobacteria*, *Actinobacteria*, and *Acinetobacter* in the conjunctival sac are associated with ocular surface irritation in patients with high myopia (≤-6D) compared to those with low myopia (>-3D). There is a positive correlation between *Acinetobacter* levels and myopic spherical equivalents. This suggests a link between conjunctival microbiota and the development of myopia [30]. Research has shown that the probiotic *Lactiplantibacillus plantarum* EP21, along with its membrane vesicles, can help prevent the progression of myopia by reducing inflammation [31].

The gut microbiome, a complex and ever-changing ecosystem of trillions of microorganisms, has a profound effect on the development of the immune system and overall physiological growth. The microbiota supplies various nutrients, regulates energy balance, modulates immune responses, and defends against pathogens [32,33]. Gut microbiome diversity undergoes rapid changes during childhood, particularly during the first 1000 days (3 years) of life, before settling into a more adult-like state [34]. In healthy individuals, the gut microbiome remains relatively stable after periods of rapid change. Nonetheless, disturbances may arise, resulting in detrimental effects on well-being and the development of diverse ailments. Dysbiosis can be broadly characterized as an asymmetry in the arrangement of gut bacteria, resulting in a deviation from customary and beneficial bacterial communities, normally present in the gastrointestinal tract [35]. Disruption of the intestinal microbiota balance can lead to a range of health issues, including digestive disorders, allergies, and chronic diseases [36–38].

Antibiotic-induced dysbiosis is associated with inflammatory responses in the eye and inflammation is a risk factor for myopia [39]. Changes in the microbiome composition and proportion of different bacteria compromise the intestinal epithelial layer, allowing microbial components to enter systemic circulation [40]. These microbe-associated molecular patterns trigger proinflammatory immune responses [40]. Numerous studies have reported a correlation between gut health and inflammatory eye diseases including glaucoma, autoimmune uveitis, diabetic retinopathy, and dry eye in patients with Sjögren’s syndrome, and age-related macular degeneration (AMD) [41].

Dysbiosis in ocular diseases such as uveitis and AMD promotes the activation of nuclear factor kappa B (NF-κB) and the NLRP3 inflammasome pathways, resulting in increased levels of tumor necrosis factor (TNF)α, interleukin (IL)-6, and IL-1β. These factors are implicated in the pathogenesis of myopia. Dysbiosis is also associated with the complement system. The complement factor H (CFH) Y402H polymorphism is associated with microbiome bacteria such as *Negativicutes*, *Clostridiales*, *Bacteroides* species, and *Ruminococcus torques*. Similar taxonomic characteristics have been observed in complement C3-deficient and wild-type mice [42]. Moreover, patients with high myopia had a significantly higher CFH concentration in the aqueous humor than patients with low myopia and in the control group. Increased CFH in the eye is associated with an increased membrane attack complex. Myopia pathogenesis has also been linked to dysregulation of the complement system. Patients with pathologic myopia have significantly elevated C3 serum concentrations (p = 0.004) and CH50 (p < 0.001) (−8 D to −25 D) [43]. Another study showed that C1q, C3, and C5b-9 levels were significantly upregulated in the sclera of negatively lens-defocused myopic guinea pigs [44]. Activation of the complement system may stimulate extracellular matrix remodeling and contribute to the development of myopia [44]. In a meta-analysis combining eight different transcriptome databases of lens-induced or form-deprivation myopia in chicks, complement system activation was found to be a biological pathway in myopia progression [45]. Thus, the eyes are highly vulnerable to complement dysregulation [45].

Dopamine in the retina has been identified as a stop signal for normal eye growth and a factor in the onset of myopia [46,47]. In animal models of form-deprivation myopia (FDM), including chicks [47,48], monkeys [49], tree shrews [50], and guinea pigs [51,52], the retinal dopamine levels were decreased. Reduced dopamine levels support the hypothesis that dopamine regulates eye growth through a signaling cascade. Therefore, dopamine inhibits FDM eye growth in rabbits [53] and dopamine agonists inhibit the progression in chicks [47,54–56]. Dopamine levels are also reduced in negative-lens-induced myopia [57], and apomorphine (a dopamine agonist) can inhibit this decrease in chickens [58,59]. Altering the precursor levels of neurotransmitters such as tryptophan or directly regulating the synthesis of neurotransmitters such as dopamine are functions of gut bacteria [60]; dysbiosis may alter dopamine levels and promote myopia.

The current study had certain limitations: 1) Information on time spent outdoors, near work activities, or parental myopia status was not available. All these factors have been suggested to play a role in the incidence of myopia. Thus, these confounders may have mitigated the effects of antibiotic exposure. Since myopia is a multifaceted condition influenced by environmental risk factors, lifestyle, and genetic variations, accounting for all confounding factors is challenging. 2) Lack of information on the use of antibiotics creams, which can be acquired from local pharmacies, to treat skin infections may have altered the gut microbiome of control subjects. 3) The retrospective study design made it difficult to establish a causal relationship between early antibiotic exposure and myopia. Thus, a large-scale prospective cohort study is warranted to confirm the association between antibiotic exposure and incidence of myopia.

These results suggest an independent relationship between antibiotic exposure during early life and incidence of myopia. Therefore, when prescribing antibiotics, physicians should be aware of the potential long-term effects of antibiotic exposure on eye health in children.

## Supplementary Information









## Figures and Tables

**Table 1:** Demographic characteristics between non-antibiotics and antibiotics cohorts.

Variables	Non-antibiotics		Antibiotics		P-value
	
	(N = 158,582)		(N = 158,582)		
	
	n	%	n	%	
Sex					0.001
Female	75,928	47.88	77,288	48.74	
Male	82,654	52.12	81,294	51.26	
Age					
Mean, (SD)^a^	1.54	0.73	1.57	0.75	0.001
Urbanization					0.01
Low	11,101	7.00	11,391	7.18	
Medium	57,156	36.04	56,400	35.57	
High	90,325	56.96	90,791	57.25	
Monthly income, (NTD)					0.01
<20,000	19,172	12.09	19,714	12.43	
20,000–40000	95,883	60.46	95,736	60.37	
>40,000	43,527	27.45	43,132	27.20	
Comorbidities					
Allergic rhinitis	14,096	8.89	14,510	9.15	0.01
Pneumonia	991	0.62	1074	0.68	0.07
Bronchitis	99,457	62.72	95,702	60.35	0.001
Sinusitis	45,954	28.98	43,511	27.44	0.001
Acute otitis media	1617	1.02	1806	1.14	0.001
Ear cellulitis	2002	1.26	2202	1.39	0.001
Pharyngitis	53,007	33.43	51,439	32.44	0.001
Tonsilitis	46,770	29.49	44,537	28.08	0.001
Laryngitis	133,998	84.50	129,206	81.48	0.001
Hordeolum	2262	1.43	2467	1.56	0.00
Gastroenteritis	26,366	16.63	26,342	16.61	0.91
Cellulitis	2023	1.28	2154	1.36	0.04
Urinary tract infection	3511	2.21	3691	2.33	0.03
Sepsis	324	0.20	383	0.24	0.03
Food allergy	46	0.03	58	0.04	0.24
Allergic conjunctivitis	6243	3.94	6535	4.12	0.01
Asthma	6027	3.80	6412	4.04	0.00
Atopic dermatitis	34,239	21.59	32,844	20.71	0.00
Follow-up mean, (SD)^a^	7.43	3.95	7.4	3.79	0.02

a Student’s t-test.

**Table 2:** The association between antibiotic exposure prior to the age of three and the risk of myopia.

Variables	Myopia			cHR	(95 % CI)	aHR†	(95 % CI)

	n	PY	IR				
Non-antibiotics	72,806	1178390.549	61.78	1.00	(Reference)	1.00	(Reference)
Antibiotics	75,302	1173232.381	64.18	1.05	(1.04, 1.06)***	1.03	(1.02, 1.04)***
Sex							
Female	74,372	1134579.882	65.55	1.00	(Reference)	1.00	(Reference)
Male	73,736	1217043.047	60.59	0.92	(0.91, 0.93)***	0.93	(0.92, 0.94)***
Urbanization							
Low	9923	177773.8973	55.82	1.00	(Reference)	1.00	(Reference)
Medium	51,789	849269.4648	60.98	1.13	(1.1, 1.15)***	1.12	(1.09, 1.14)***
High	86,396	1324579.567	65.23	1.23	(1.2, 1.25)***	1.19	(1.17, 1.22)***
Monthly income, (NTD)							
<20,000	16,073	322753.9932	49.80	1.00	(Reference)	1.00	(Reference)
20,000–40000	91,442	1417098.839	64.53	1.38	(1.36, 1.41)***	1.39	(1.36, 1.41)***
>40,000	40,593	611770.0972	66.35	1.46	(1.43, 1.49)***	1.46	(1.43, 1.49)***
Comorbidities							
Allergic rhinitis	12,555	187831.8	66.84	1.15	(1.13, 1.17)***	1.04	(1.02, 1.06)***
Pneumonia	639	11264.96	56.72	1.08	(1, 1.17)	0.94	(0.87, 1.01)
Bronchitis	88,079	1,398,011	63.00	1.05	(1.03, 1.06)***	1.00	(0.99, 1.01)
Sinusitis	34,676	601272.1	57.67	0.95	(0.94, 0.97)***	0.88	(0.87, 0.89)***
Acute otitis media	1481	22537.94	65.71	1.11	(1.06, 1.17)***	0.99	(0.94, 1.05)
Ear cellulitis	1873	30123.43	62.18	1	(0.96, 1.05)	0.96	(0.92, 1.01)
Pharyngitis	44,131	717596.9	61.50	1.03	(1.02, 1.04)***	0.96	(0.95, 0.97)***
Tonsilitis	40,230	623386.8	64.53	1.1	(1.09, 1.11)***	1.00	(0.99, 1.01)
Laryngitis	121,328	1,936,939	62.64	0.99	(0.98, 1.01)	0.94	(0.93, 0.96)***
Hordeolum	2123	31320.22	67.78	1.15	(1.1, 1.2)***	0.95	(0.91, 0.99)*
Gastroenteritis	24,701	375892.8	65.71	1.08	(1.06, 1.09)***	1.03	(1.02, 1.05)***
Cellulitis	1947	28575.36	68.14	1.14	(1.09, 1.19)***	1.07	(1.03, 1.12)**
Urinary tract infection	3327	47588.36	69.91	1.19	(1.15, 1.23)***	1.12	(1.08, 1.16)***
Sepsis	416	5506.73	75.54	1.17	(1.06, 1.29)**	1.09	(0.99, 1.20)
Food allergy	45	761.6208	59.08	0.92	(0.69, 1.24)	0.89	(0.66, 1.19)
Allergic conjunctivitis	6253	87402.56	71.54	1.21	(1.18, 1.24)***	1.09	(1.07, 1.12)***
Asthma	6337	85184.85	74.39	1.24	(1.21, 1.27)***	1.08	(1.05, 1.11)***
Atopic dermatitis	25,285	445646.4	56.74	0.95	(0.94, 0.96)***	0.94	(0.92, 0.95)***

PY: person-years; IR: incidence rate per 1000 person-years; cHR: crude hazard ratio; aHR: adjusted hazard ratio.

† Adjusted by sex, age, urbanization, monthly income, comorbidities.

* p-value <0.05;

** p < 0.01,

*** p < 0.001.

**Table 3:** Stratification of the association between antibiotic exposure prior to the age of three and the risk of myopia by demographics and comorbidities.

Variable	Non-antibiotics			Antibiotics			Crude			Adjusted		
			
	n	PY	IR	n	PY	IR	cHR	(95 % CI)	p-value	aHR	(95 % CI)	p-value
Sex												
Female	36,122	563,542	64.10	38,250	571,037	66.98	1.05	(1.04, 1.07)***	<0.001	1.05	(1.04, 1.07)***	<0.001
Male	36,684	614,848	59.66	37,052	602,195	61.53	1.04	(1.02, 1.05)***	<0.001	1.04	(1.02, 1.05)***	<0.001
Urbanization												
Low	4914	88,389	55.60	5009	89,385	56.04	1.02	(0.98, 1.06)	0.2788	1.02	(0.98, 1.06)	0.2968
Medium	25,726	428,660	60.01	26,063	420,610	61.96	1.04	(1.02, 1.06)***	<0.001	1.04	(1.02, 1.06)***	<0.001
High	42,166	661,342	63.76	44,230	663,238	66.69	1.05	(1.04, 1.07)***	<0.001	1.05	(1.04, 1.06)***	<0.001
Monthly income, (NTD)												
<20,000	7596	161,780	46.95	8477	160,974	52.66	1.14	(1.11, 1.18)***	<0.001	1.14	(1.11, 1.18)***	<0.001
20,000–40000	44,947	709,670	63.34	46,495	707,428	65.72	1.04	(1.03, 1.06)***	<0.001	1.04	(1.03, 1.05)***	<0.001
>40,000	20,263	306,941	66.02	20,330	304,829	66.69	1.01	(0.99, 1.03)	0.1676	1.01	(0.99, 1.03)	0.2461
Comorbidities												
Allergic rhinitis												
No	66,378	1,085,208	61.17	69,175	1,078,583	64.14	1.06	(1.04, 1.07)***	<0.001	1.05	(1.04, 1.06)***	<0.001
Yes	6428	93,182	68.98	6127	94,649	64.73	0.95	(0.91, 0.98)^**^	0.0021	0.95	(0.91, 0.98)^**^	0.0025
Pneumonia												
No	72,468	1,172,692	61.80	75,001	1,167,665	64.23	1.05	(1.04, 1.06)***	<0.001	1.04	(1.03, 1.05)***	<0.001
Yes	338	5698	59.32	301	5567	54.07	0.99	(0.85, 1.16)	0.9052	0.97	(0.82, 1.14)	0.6877
Bronchitis												
No	27,127	463,181	58.57	32,902	490,430	67.09	1.16	(1.14, 1.18)***	<0.001	1.15	(1.13, 1.17)***	<0.001
Yes	45,679	715,209	63.87	42,400	682,802	62.10	0.98	(0.96, 0.99)***	<0.001	0.97	(0.96, 0.99)***	<0.001
Sinusitis												
No	54,155	867,038	62.46	59,277	883,313	67.11	1.08	(1.07, 1.09)***	<0.001	1.08	(1.07, 1.09)***	<0.001
Yes	18,651	311,352	59.90	16,025	289,920	55.27	0.93	(0.91, 0.95)***	<0.001	0.94	(0.92, 0.96)***	<0.001
Acute otitis media												
No	72,063	1,167,176	61.74	74,564	1,161,909	64.17	1.05	(1.04, 1.06)***	<0.001	1.04	(1.03, 1.05)***	<0.001
Yes	743	11,215	66.25	738	11,323	65.17	1.03	(0.93, 1.14)	0.5881	1.04	(0.94, 1.16)	0.4321
Ear cellulitis												
No	71,810	1,163,421	61.72	74,425	1,158,078	64.27	1.05	(1.04, 1.06)***	<0.001	1.05	(1.03, 1.06)***	<0.001
Yes	996	14,970	66.54	877	15,154	57.87	0.9	(0.82, 0.98)*	0.0207	0.91	(0.83, 1)	0.0548
Pharyngitis												
No	49,770	812,206	61.28	54,207	821,820	65.96	1.08	(1.07, 1.1)***	<0.001	1.08	(1.07, 1.09)***	<0.001
Yes	23,036	366,185	62.91	21,095	351,412	60.03	0.96	(0.94, 0.98)***	<0.001	0.96	(0.94, 0.98)***	<0.001
Tonsilitis												
No	51,496	856,615	60.12	56,382	871,621	64.69	1.08	(1.07, 1.1)***	<0.001	1.08	(1.07, 1.09)***	<0.001
Yes	21,310	321,775	66.23	18,920	301,612	62.73	0.96	(0.94, 0.97)***	<0.001	0.95	(0.93, 0.97)***	<0.001
Laryngitis												
No	10,481	191,995	54.59	16,299	222,689	73.19	1.39	(1.36, 1.43)***	<0.001	1.35	(1.32, 1.39)***	<0.001
Yes	62,325	986,396	63.18	59,003	950,543	62.07	0.99	(0.97, 1)^**^	0.0098	0.99	(0.98, 1)*	0.0195
Hordeolum												
No	71,674	1,163,045	61.63	74,311	1,157,257	64.21	1.05	(1.04, 1.06)***	<0.001	1.05	(1.04, 1.06)***	<0.001
Yes	1132	15,345	73.77	991	15,975	62.03	0.85	(0.78, 0.93)***	<0.001	0.86	(0.79, 0.94)***	<0.001
Gastroenteritis												
No	60,038	988,960	60.71	63,369	986,770	64.22	1.06	(1.05, 1.08)***	<0.001	1.06	(1.05, 1.07)***	<0.001
Yes	12,768	189,430	67.40	11,933	186,462	64.00	0.96	(0.93, 0.98)***	<0.001	0.96	(0.93, 0.98)***	<0.001
Cellulitis												
No	71,828	1,164,036	61.71	74,333	1,159,012	64.13	1.05	(1.04, 1.06)***	<0.001	1.04	(1.03, 1.05)***	<0.001
Yes	978	14,355	68.13	969	14,221	68.14	1.04	(0.95, 1.14)	0.3705	1.03	(0.95, 1.13)	0.4625
Urinary tract infection												
No	71,242	1,154,893	61.69	73,539	1,149,142	63.99	1.04	(1.03, 1.05)***	<0.001	1.04	(1.03, 1.05)***	<0.001
Yes	1564	23,498	66.56	1763	24,090	73.18	1.13	(1.06, 1.21)***	<0.001	1.11	(1.04, 1.19)^**^	0.0022
Allergic conjunctivitis												
No	69,493	1,135,235	61.21	72,362	1,128,986	64.09	1.05	(1.04, 1.07)***	<0.001	1.05	(1.04, 1.06)***	<0.001
Yes	3313	43,156	76.77	2940	44,247	66.45	0.87	(0.83, 0.92)***	<0.001	0.87	(0.83, 0.91)***	<0.001
Asthma												
No	69,680	1,135,851	61.35	72,091	1,130,587	63.76	1.05	(1.03, 1.06)***	<0.001	1.04	(1.03, 1.05)***	<0.001
Yes	3126	42,539	73.49	3211	42,646	75.29	1.06	(1.01, 1.11)*	0.0231	1.02	(0.97, 1.07)	0.5016
Atopic dermatitis												
No	59,280	950,336	62.38	63,543	955,641	66.49	1.07	(1.06, 1.09)***	<0.001	1.07	(1.06, 1.08)***	<0.001
Yes	13,526	228,055	59.31	11,759	217,592	54.04	0.92	(0.89, 0.94)***	<0.001	0.92	(0.9, 0.95)***	<0.001

PY: person-years; IR: incidence rate per 1000 person-years; cHR: crude hazard ratio; aHR: adjusted hazard ratio.

† Adjusted by sex, age, urbanization, monthly income, comorbidities.

* p < 0.05;

*** p < 0.001.

**Table 4:** Hazard ratio of myopia for stratification by the duration of antibiotic drug use days.

Variables	Myopia			cHR	(95 % CI)	aHR	(95 % CI)

	n	PY	IR				
Non use of Antibiotics	72,806	1,178,391	61.78	1.00	(Reference)	1.00	(Reference)
Any antibiotics							
<11	3641	65,028	55.99	1.18	(1.14, 1.22)^**^	1.19	(1.15, 1.23)^**^
11–80	50,575	818,011	61.83	1.02	(1.01, 1.04)^**^	1.02	(1.01, 1.04)^**^
≧80	21,086	290,193	72.66	1.08	(1.06, 1.1)***	1.07	(1.05, 1.08)^**^
First-generation cephalosporins							
<8	1906	31,318	60.86	1.01	(0.96, 1.05)	1.00	(0.96, 1.05)
8–32	31,109	479,234	64.91	1.00	(0.99, 1.01)	1.00	(0.99, 1.02)
≧32	10,788	142,285	75.82	1.13	(1.1, 1.15)***	1.12	(1.1, 1.14)***
Penicillins with extended spectrum							
<9	3117	45,735	68.15	1.15	(1.11, 1.2)***	1.15	(1.11, 1.2)***
9–43	40,651	649,715	62.57	1.02	(1, 1.03)^**^	1.01	(1, 1.03)^*^
≧43	12,930	206,313	62.67	0.95	(0.93, 0.96)^**^	0.94	(0.92, 0.95)^**^
Others							
<8	327	5840	55.99	1.18	(1.06, 1.32)^**^	1.19	(1.07, 1.33)^**^
8–23	5898	92,619	63.68	1.18	(1.15, 1.21)^**^	1.19	(1.15, 1.22)^**^
≧23	2458	30,837	79.71	1.34	(1.29, 1.4)***	1.35	(1.3, 1.41)***

PY: person-years; IR: incidence rate per 1000 person-years; cHR: crude hazard ratio; aHR: adjusted hazard ratio.

† Adjusted by sex, age, urbanization, monthly income, comorbidities.

*** p < 0.001.

**Table 5:** Hazard ratio of myopia for stratification by the cumulative define daily dose (DDD) of antibiotics.

Variables	Myopia			cHR	(95 % CI)	aHR	(95 % CI)

	n	PY	IR				
Non use of Antibiotics	72,806	1,178,391	61.78	1.00	(Reference)	1.00	(Reference)
Any antibiotics							
<2833	15,400	229,030	67.24	1.3	(1.28, 1.32)**	1.31	(1.28, 1.33)**
2833–6083	17,629	272,575	64.68	1.11	(1.09, 1.12)**	1.11	(1.09, 1.13)**
≧6083	42,273	671,627	62.94	0.96	(0.95, 0.97)**	0.95	(0.94, 0.96)**
First-generation cephalosporins							
<5625	9413	130,081	72.36	1.25	(1.23, 1.28)**	1.26	(1.24, 1.29)**
5625–10134	11,017	160,820	68.51	1.07	(1.05, 1.09)**	1.07	(1.05, 1.09)**
≧10,134	23,373	361,936	64.58	0.95	(0.93, 0.96)**	0.94	(0.93, 0.95)**
Penicillins with extended spectrum							
<4042	10,868	170,380	63.79	1.21	(1.19, 1.24)**	1.22	(1.19, 1.24)**
4042–7833	13,667	213,762	63.94	1.06	(1.04, 1.08)**	1.06	(1.04, 1.08)**
≧7833	32,163	517,621	62.14	0.93	(0.92, 0.94)**	0.93	(0.91, 0.94)**
Others							
<875	2759	36,680	75.22	1.48	(1.43, 1.54)**	1.5	(1.44, 1.56)**
875–1500	1848	25,390	72.78	1.38	(1.31, 1.44)**	1.39	(1.33, 1.46)**
≧1500	4076	67,226	60.63	1.04	(1.01, 1.08)*	1.05	(1.01, 1.08)**

PY: person-years; IR: incidence rate per 1000 person-years; cHR: crude hazard ratio; aHR: adjusted hazard ratio.

† Adjusted by sex, age, urbanization, monthly income, comorbidities.

* p < 0.05;

** p < 0.01;

*** p < 0.001.
