# Supplementary figures and images for: Early-life exposure to antibiotics increases the risk of myopia: A retrospective cohort study
Source: Biomedicine (Taipei). 2026 Jun 1;16(2):75–86. doi: 10.37796/2211-8039.1666 (PMC13387399; doi:10.37796/2211-8039.1666)

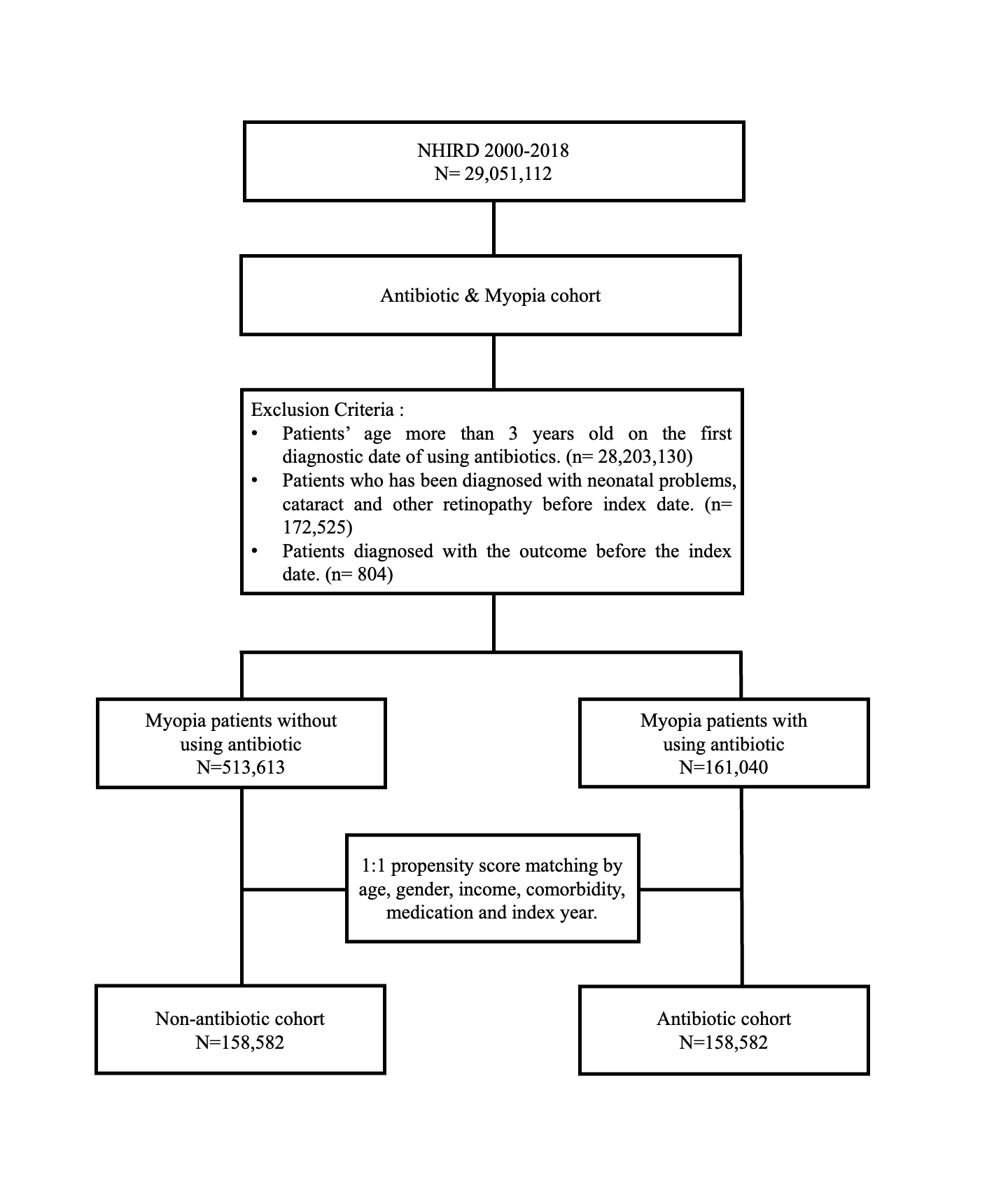

Supplement: Supplementary file 1 [file bmed-16-02-075-g001.tiff]
