# Supplementary material for: Early-life exposure to antibiotics increases the risk of myopia: A retrospective cohort study
Source: Biomedicine (Taipei). 2026 Jun 1;16(2):75–86. doi: 10.37796/2211-8039.1666 (PMC13387399; doi:10.37796/2211-8039.1666)
Supplement: Supplementary file 2 [file bmed-16-02-075-s001.docx]

| Supplementary Table 1. Demographic characteristics between non-antibiotics and antibiotics cohorts | | | | | |
| --- | --- | --- | --- | --- | --- |
|  | Non-antibiotics | | Antibiotics | |  |
|  | (N=513613) | | (N=161040) | |  |
| Variables | n | % | n | % | P-value |
| Sex |  |  |  |  | 0.82 |
| female | 249368 | 48.55 | 78241 | 48.58 |  |
| male | 264245 | 51.45 | 82799 | 51.42 |  |
| Age |  |  |  |  |  |
| mean, (SD) † | 1.99 | 0.82 | 1.56 | 0.75 | <0.001 |
| Urbanization |  |  |  |  | <0.001 |
| low | 37692 | 7.34 | 11512 | 7.15 |  |
| medium | 181386 | 35.32 | 57437 | 35.67 |  |
| high | 294535 | 57.35 | 92091 | 57.19 |  |
| Monthly income, (NTD) | |  |  |  | <0.001 |
| <20000 | 69744 | 13.58 | 19714 | 12.24 |  |
| 20000-40000 | 302287 | 58.86 | 97640 | 60.63 |  |
| >40000 | 141582 | 27.57 | 43686 | 27.13 |  |
| Comorbidities |  |  |  |  |  |
| Allergic rhinitis | 80516 | 15.68 | 14528 | 9.02 | <0.001 |
| Pneumonia | 10869 | 2.12 | 1074 | 0.67 | <0.001 |
| Bronchitis | 376497 | 73.30 | 95768 | 59.47 | <0.001 |
| Sinusitis | 243485 | 47.41 | 43511 | 27.02 | <0.001 |
| Acute otitis media | 16388 | 3.19 | 1806 | 1.12 | <0.001 |
| Ear cellulitis | 13849 | 2.70 | 2202 | 1.37 | <0.001 |
| Pharyngitis | 229664 | 44.72 | 51481 | 31.97 | <0.001 |
| Tonsilitis | 231986 | 45.17 | 44537 | 27.66 | <0.001 |
| Laryngitis | 447885 | 87.20 | 130694 | 81.16 | <0.001 |
| Hordeolum | 16885 | 3.29 | 2467 | 1.53 | <0.001 |
| Gastroenteritis | 122561 | 23.86 | 26347 | 16.36 | <0.001 |
| Cellulitis | 19736 | 3.84 | 2154 | 1.34 | <0.001 |
| Urinary tract infection | 34662 | 6.75 | 3691 | 2.29 | <0.001 |
| Sepsis | 3875 | 0.75 | 383 | 0.24 | <0.001 |
| Food allergy | 277 | 0.05 | 58 | 0.04 | <0.001 |
| Allergic conjunctivitis | 36064 | 7.02 | 6535 | 4.06 | <0.001 |
| Asthma | 41887 | 8.16 | 6412 | 3.98 | <0.001 |
| Atopic dermatitis | 131752 | 25.65 | 33012 | 20.50 | <0.001 |
| Follow-up mean, (SD) † | 6.38 | 3.96 | 7.41 | 3.78 | <.0001 |

†: Student’s t-test; Chi-square test
