# Supplementary material for: Early-life exposure to antibiotics increases the risk of myopia: A retrospective cohort study
Source: Biomedicine (Taipei). 2026 Jun 1;16(2):75–86. doi: 10.37796/2211-8039.1666 (PMC13387399; doi:10.37796/2211-8039.1666)
Supplement: Supplementary file 3 [file bmed-16-02-075-s002.docx]

| Supplementary Table 2. The association between antibiotic exposure prior to the age of three and the risk of myopia | | | | | | | |
| --- | --- | --- | --- | --- | --- | --- | --- |
|  | Myopia | | |  |  |  |  |
| Variables | n | PY | IR | cHR | (95% CI) | aHR | (95% CI) |
| Non-antibiotics | 200483 | 3276529 | 61.19 | 1.00 | (reference) | 1.00 | (reference) |
| Antibiotics | 77746 | 1193325 | 65.15 | 1.01 | (1, 1.02)* | 1.14 | (1.13, 1.15)*** |
| Sex |  |  |  |  |  |  |  |
| female | 139139 | 2163464 | 64.31 | 1.00 | (reference) | 1.00 | (reference) |
| male | 139090 | 2306389 | 60.31 | 0.93 | (0.93, 0.94)*** | 0.93 | (0.93, 0.94)*** |
| Urbanization |  |  |  |  |  |  |  |
| low | 19019 | 349078.2 | 54.48 | 1.00 | (reference) | 1.00 | (reference) |
| medium | 96976 | 1602096 | 60.53 | 1.14 | (1.13, 1.16)*** | 1.13 | (1.11, 1.15)*** |
| high | 162234 | 2518679 | 64.41 | 1.24 | (1.22, 1.26)*** | 1.20 | (1.19, 1.22)*** |
| Monthly income, (NTD) |  |  |  |  |  |  |  |
| <20000 | 31969 | 668255.5 | 47.84 | 1.00 | (reference) | 1.00 | (reference) |
| 20000-40000 | 170269 | 2647364 | 64.32 | 1.43 | (1.41, 1.44)*** | 1.42 | (1.40, 1.43)*** |
| >40000 | 75991 | 1154234 | 65.84 | 1.51 | (1.49, 1.53)*** | 1.50 | (1.48, 1.52)*** |
| Comorbidities |  |  |  |  |  |  |  |
| Allergic rhinitis | 38725 | 574339.9 | 67.43 | 1.16 | (1.15, 1.17)*** | 1.03 | (1.02, 1.04)*** |
| Pneumonia | 3636 | 61790.27 | 58.84 | 1.07 | (1.04, 1.11)*** | 0.93 | (0.90, 0.96)*** |
| Bronchitis | 197281 | 3074848 | 64.16 | 1.14 | (1.13, 1.15)*** | 1.02 | (1.01, 1.03)*** |
| Sinusitis | 112174 | 1793556 | 62.54 | 1.06 | (1.05, 1.07)*** | 0.92 | (0.92, 0.93)*** |
| Acute otitis media | 7402 | 111556.2 | 66.35 | 1.11 | (1.08, 1.14)*** | 0.99 | (0.97, 1.02) |
| Ear cellulitis | 6874 | 104372.5 | 65.86 | 1.07 | (1.05, 1.1)*** | 1.00 | (0.97, 1.02) |
| Pharyngitis | 113476 | 1779348 | 63.77 | 1.09 | (1.08, 1.1)*** | 0.98 | (0.97, 0.99)*** |
| Tonsilitis | 116798 | 1759746 | 66.37 | 1.16 | (1.15, 1.17)*** | 1.03 | (1.02, 1.04)*** |
| Laryngitis | 242900 | 3829459 | 63.43 | 1.17 | (1.15, 1.18)*** | 1.03 | (1.01, 1.04)*** |
| Hordeolum | 8481 | 119437.2 | 71.01 | 1.19 | (1.16, 1.22)*** | 1.00 | (0.98, 1.03) |
| Gastroenteritis | 65792 | 969122.6 | 67.89 | 1.13 | (1.12, 1.14)*** | 1.06 | (1.05, 1.07)*** |
| Cellulitis | 9215 | 140209.6 | 65.72 | 1.08 | (1.06, 1.1)*** | 0.99 | (0.97, 1.02) |
| Urinary tract infection | 15010 | 229227.8 | 65.48 | 1.11 | (1.09, 1.13)*** | 1.04 | (1.03, 1.06)*** |
| Sepsis | 2004 | 29749.39 | 67.36 | 1.06 | (1.02, 1.11)** | 1.01 | (0.96, 1.05) |
| Food allergy | 122 | 2100.758 | 58.07 | 0.93 | (0.78, 1.12) | 0.85 | (0.71, 1.02) |
| Allergic conjunctivitis | 19681 | 261165.7 | 75.36 | 1.3 | (1.28, 1.32)*** | 1.15 | (1.14, 1.17)*** |
| Asthma | 21648 | 304423.7 | 71.11 | 1.19 | (1.17, 1.2)*** | 1.04 | (1.03, 1.06)*** |
| Atopic dermatitis | 57962 | 980676.8 | 59.10 | 1 | (0.99, 1.01) | 0.95 | (0.94, 0.96)*** |

PY: person-years; IR: incidence rate per 1,000 person-years; cHR: crude hazard ratio; aHR: adjusted hazard ratio;

†: adjusted by sex, age, urbanization, monthly income, comorbidities

*: p-value<0.05; **p<0.01, ***p<0.001
