# Supplementary material for: Early-life exposure to antibiotics increases the risk of myopia: A retrospective cohort study
Source: Biomedicine (Taipei). 2026 Jun 1;16(2):75–86. doi: 10.37796/2211-8039.1666 (PMC13387399; doi:10.37796/2211-8039.1666)
Supplement: Supplementary file 4 [file bmed-16-02-075-s003.docx]

| Supplememtary Table 3. The frequency of antibiotic use in this study | |  |
| --- | --- | --- |
| Antibiotics | Frequency (%) | |
| Penicillins with extended spectrum | 32.5 | |
| First-generation cephalosporins | 26.7 | |
| Second-generation cephalosporins | 7.4 | |
| Combinations of sulfonamides and trimethoprim, incl. derivatives | 6.57 | |
| Macrolides | 5.9 | |
| Tetracyclines | 3.31 | |
| Combinations of penicillins, incl. beta-lactamase inhibitors | 3.08 | |
| Lincosamides | 2.43 | |
| Amphenicols | 2.26 | |
| Fluoroquinolones | 1.61 | |
| Third-generation cephalosporins | 1.03 | |
| Trimethoprim and derivatives | 0.82 | |
| Beta-lactamase sensitive penicillins | 0.78 | |
| Other quinolones | 0.49 | |
| Other aminoglycosides | 0.26 | |
| Other antibacterials | 0.04 | |
| Imidazole derivatives | 0.005 | |
| Nitrofuran derivatives | 0.004 | |
| Glycopeptide antibacterials | 0.002 | |
| Long-acting sulfonamides | 0.002 | |
| Fourth-generation cephalosporins | 0.0003 | |
| Polymyxins | 0.0003 | |
